# Bibliometric and Latent Dirichlet Allocation (LDA) analysis of artificial intelligence for thyroid nodules

**DOI:** 10.3389/fendo.2026.1932519

**Published:** 2026-09-10

**Authors:** Xinxin Li, Jiliang Yan, Na Li, Minghui Li, Yanjie Han

**Affiliations:** 1 Department of Laboratory Medicine, The Second People’s Hospital of Lianyungang, Haizhou District, Lianyungang, Jiangsu, China; 2 Department of Clinical Laboratory, Ningbo Medical Center Lihuili Hospital, The Affiliated Lihuili Hospital of Ningbo University, Ningbo, China; 3 The Second People’s Hospital of Lianyungang Affiliated to Kangda College of Nanjing Medical University, Lianyungang, Jiangsu, China

**Keywords:** artificial intelligence, bibliometric analysis, latent dirichlet allocation, research trends, thyroid nodules

## Abstract

**Background:**

Artificial intelligence (AI) has rapidly integrated into thyroid nodule ultrasound diagnosis and malignant risk stratification, fostering a fast-growing interdisciplinary domain encompassing endocrinology, medical imaging, and computational intelligence. Nevertheless, existing bibliometric studies in this field rely on single-database datasets and short time-series windows, lacking systematic elaboration of the dynamic linkage between clinical guideline iterations and AI algorithm evolution. This study conducted a comprehensive bibliometric analysis to fill this research gap, clarify the global intellectual landscape, and reveal the evolutionary rules and emerging frontiers of AI-assisted thyroid nodule diagnosis.

**Methods:**

Peer-reviewed articles and reviews on AI in thyroid nodule diagnosis were retrieved from Web of Science and PubMed (2006-2025). After strict data cleaning, 821 valid publications were analyzed using CiteSpace, VOSviewer, and online platforms, examining publication trends, contributions, collaboration networks, citations, and keyword evolution. Latent Dirichlet Allocation (LDA) was applied for topic modeling.

**Results:**

The field showed rapid and sustained growth, with a compound annual growth rate of 24.1%, and a notable acceleration around 2017. In total, 2,870 authors from 75 countries contributed. China led global output, while the United States and South Korea showed higher citation quality. Shanghai Jiao Tong University was the most productive institution. Keyword analysis revealed close coupling with clinical guidelines (ATA, TI-RADS, Bethesda). Core hotspots included ultrasound image analysis, deep learning optimization, and malignant risk stratification. Emerging frontiers involve explainable AI, multimodal data fusion, and standardized clinical validation.

**Conclusions:**

This study establishes a long-time-series, multi-database knowledge map of AI applications in thyroid nodule diagnosis, systematically revealing the global research pattern, spatiotemporal evolution characteristics, and guideline-driven developmental logic of this field. Different from previous single-dimensional bibliometric summaries, this work clarifies how standardized clinical diagnostic criteria chronologically correspond with the iterative progress of radiomics and deep learning research. The findings provide reliable theoretical references for subsequent algorithm optimization, interdisciplinary innovation, and large-scale multicenter prospective validation, facilitating the standardized clinical translation and high-quality development of AI-assisted thyroid diagnostic models and precision endocrinology.

## Introduction

Thyroid nodules represent one of the most frequently encountered abnormalities in clinical endocrinology, with a detection rate exceeding 60% in the general population when assessed by high-resolution ultrasound (1–4). Although the vast majority are benign, the global rise in thyroid cancer incidence has intensified the need for accurate risk stratification and timely diagnosis (5, 6). The morphological diversity of thyroid nodules, combined with the substantial overlap in imaging characteristics between benign and malignant lesions, presents a persistent diagnostic challenge (7, 8). Conventional diagnostic modalities—including ultrasound, fine-needle aspiration biopsy, and molecular testing—have markedly improved diagnostic accuracy; nonetheless, they remain constrained by inter-observer variability, procedural invasiveness, and the inherent complexity of thyroid pathology (9–11). These limitations underscore the urgent demand for more precise, non-invasive, and efficient tools to support clinical decision-making in thyroid nodule management.

In recent years, AI has emerged as a transformative force in medical imaging and diagnostic medicine (12, 13). By harnessing advanced techniques such as machine learning, deep learning, and radiomics, AI enables the automated extraction of high-dimensional imaging features that often elude human perception (14, 15). Within the domain of thyroid nodules, AI-based models have shown considerable promise in enhancing ultrasound image interpretation, improving diagnostic accuracy, and reducing unnecessary invasive procedures (16–18). Emerging evidence suggests that deep learning algorithms can achieve diagnostic performance comparable to, or in some instances surpassing, that of experienced radiologists, particularly in distinguishing benign from malignant nodules (19, 20). Moreover, the integration of AI with clinical parameters, genomic profiles, and multimodal imaging has opened new avenues for personalized risk stratification and therapeutic planning (21–23). Despite the rapid expansion of AI-related research in this field, the literature remains fragmented, with limited synthesis of global research trajectories, key contributors, and emerging frontiers (24–26).

Bibliometric analysis provides a systematic and quantitative framework for evaluating scientific literature, facilitating the objective identification of research trends, influential publications, leading institutions, and collaborative networks within a given domain (27, 28). By leveraging bibliometric tools such as CiteSpace and VOSviewer, researchers can map the intellectual structure of a discipline, detect citation bursts, and trace the evolution of research hotspots over time (29, 30). Several pioneering bibliometric studies have outlined the research landscape of AI in thyroid nodule diagnosis. Among them, Peng et al. (2023) performed the first systematic analysis based on 601 publications from 1996 to 2023, identifying China as the leading contributor and highlighting radiomics, deep learning, and central lymph node metastasis (CLNM) as major research hotspots (31). Nevertheless, previous bibliometric work including Peng et al. (2023) relied solely on publications retrieved from the Web of Science Core Collection with a relatively short observation window (1996-2023); these studies are further constrained by single-dimensional statistical descriptions and insufficient integration between the evolution of clinical guidelines and advances in AI algorithms. Few studies have established a clear chronological link between iterations of ATA, TI-RADS, and Bethesda criteria and the shifting trends of deep learning and radiomics research, leading to a gap between technical innovation and real-world clinical application. Against this background, a comprehensive bibliometric analysis connecting the evolution of clinical guidelines with algorithm development is urgently needed to provide a clear knowledge map for researchers, clinicians, and policymakers, thereby guiding future investigation and facilitating interdisciplinary collaboration (1, 32, 33). This study aims to conduct a comprehensive bibliometric analysis of the application of artificial intelligence in thyroid nodule research, covering all relevant literature from 2006 to 2025. Academic documents were retrieved from the Web of Science Core Collection and analyzed using CiteSpace, VOSviewer, and an online bibliometric platform (34–36). Through systematic examination of publication trends, author productivity, institutional contributions, journal distribution, geographic patterns, and thematic structures, this study seeks to construct a systematic knowledge map of the field, identify key research frontiers, and establish a dynamic linkage between ATA/TI-RADS guideline iteration and deep learning/radiomics algorithm advancement. The findings are intended to serve as a valuable reference for researchers seeking to understand the developmental trajectory, current hotspots, and future directions of AI applications in thyroid nodule diagnosis, and to provide directional support for the clinical translation of AI-assisted diagnostic models. The main novelties of the present study are summarized as follows:

1. It constructs a longer time-series bibliometric framework covering two decades (2006-2025) to capture the latest research frontiers without affecting the rigor of overall trend analysis.
2. It systematically links the iteration of core clinical guidelines (ATA, TI-RADS, Bethesda) with the evolution of AI research hotspots, clarifying how the iterative updates of clinical guidelines chronologically correspond with the evolving phases of deep learning and radiomics research.
3. Compared with previous datasets of limited scale (e.g., 601 publications in prior studies), this study adopted an expanded dataset of 821 records with strict standardized data cleaning (author/institution disambiguation, duplicate removal, strict document type filtering), providing a more comprehensive and stable knowledge mapping for this interdisciplinary field.

This study might assist clinicians and engineers in identifying unmet clinical needs and prioritizing future directions for the clinical translation of AI models.

## Materials and methods

### Data source and retrieval strategy

The Web of Science Core Collection (WoSCC) and Pubmed database were selected as the primary data source for this bibliometric study. WoSCC and Pubmed are recognized as two of the most authoritative and comprehensive academic databases worldwide, ensuring the reliability and representativeness of the retrieved publications (37–39). Additionally, it provides detailed and standardized bibliographic records, facilitating subsequent analyses such as knowledge mapping and collaboration visualization (40–42).

To ensure the accuracy and comprehensiveness of literature retrieval, a topic search was conducted in WoSCC and Pubmed using a combination of MeSH (Medical Subject Headings) terms and Entry Terms. MeSH serves as a standardized controlled vocabulary for indexing biomedical literature, while Entry Terms encompass synonymous and closely related expressions that further enhance search sensitivity (43, 44).

All retrieval operations across the two databases were uniformly completed on June 01, 2026, Beijing Time. The whole search procedure was continuously executed within 9:00-17:00 on the same day without multi-day batch retrieval. All retrieval operations across the two databases were uniformly completed on June 01, 2026, Beijing Time, between 9:00 AM and 5:00 PM, without multi-day batch retrieval. This procedural control minimized the risk of incremental database updates during the search window and ensured that the search snapshots from the two databases remained synchronized. The publication time frame was strictly limited to 2006–2025 through the search criteria.

Separate retrieval queries were established for Web of Science Core Collection (WoSCC) and PubMed according to the indexing rules of each database. For WoSCC, topic search (TS) was used for retrieval, and the complete retrieval formula is shown below: TS = (“thyroid nodule” OR “thyroid nodules”) AND (“artificial intelligence” OR “AI” OR “deep learning” OR “machine learning” OR “CNN” OR “radiomics” OR “computational intelligence” OR “convolutional neural network”). For PubMed, a hybrid search strategy combining MeSH controlled terms and free-text terms from titles and abstracts was adopted. The PubMed full search query is as follows: ((“thyroid nodule”[MeSH Terms] OR thyroid nodules[Title/Abstract]) AND (“Artificial Intelligence”[MeSH Terms] OR deep learning[Title/Abstract] OR machine learning[Title/Abstract] OR CNN[Title/Abstract] OR radiomics[Title/Abstract] OR “convolutional neural network”[Title/Abstract])).

**Figure 1:**
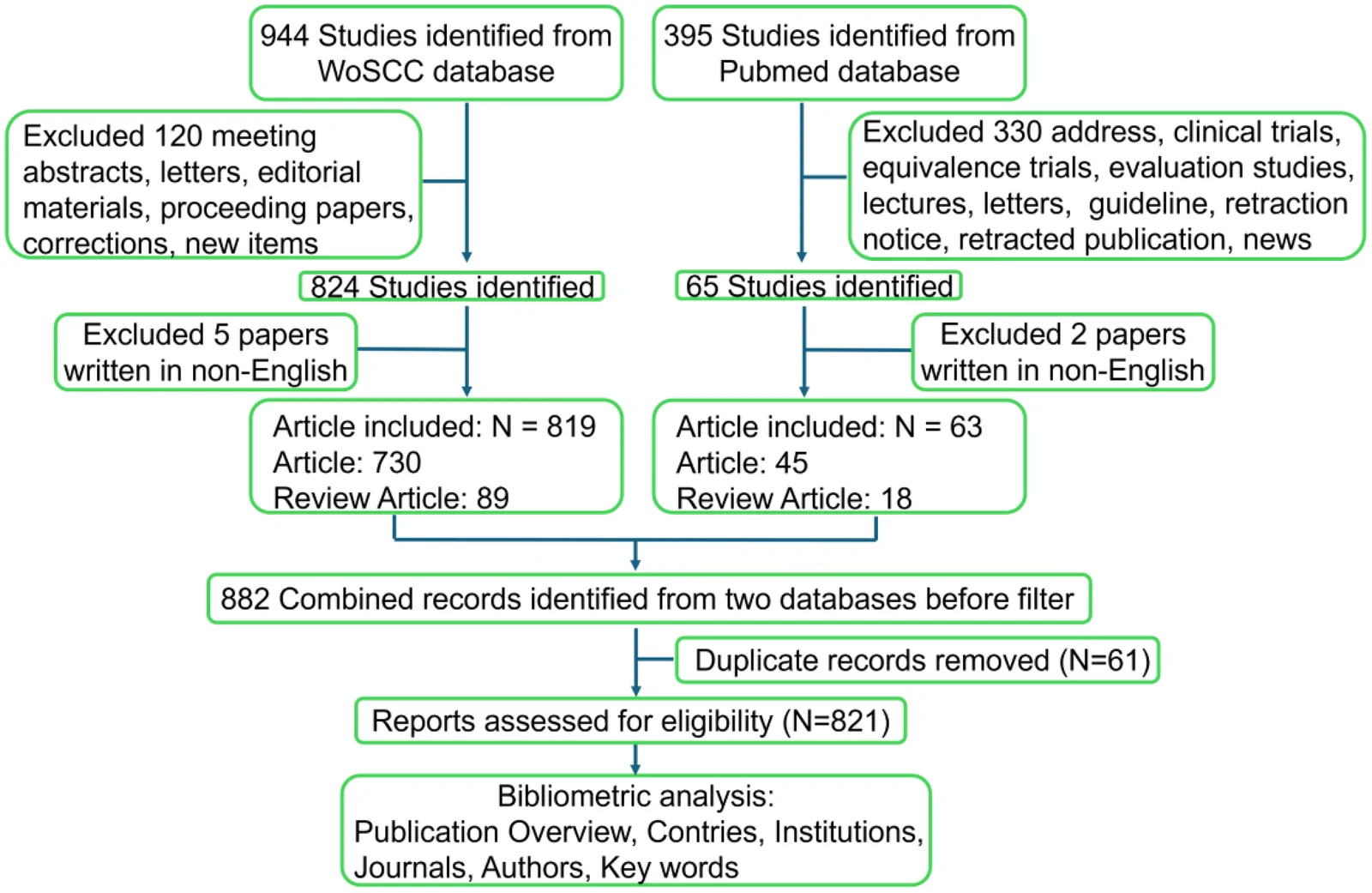
PRISMA flow diagram for literature screening from the WoSCC and PubMed databases.

The inclusion criteria were: publication types limited to “article” or “review article”; and English-language publications. The initial combined search across both databases returned 882 records prior to any filtering. After applying the inclusion/exclusion criteria, 61 non-eligible records (e.g., conference abstracts, letters, editorials, and case reports) were excluded. A total of 821 records were identified in the initial search phase. All retrieved terms were standardized with MeSH entry terms to optimize retrieval sensitivity, while duplicate records were removed via EndNote combined with manual second-round proofreading. After deduplication, 61 duplicate entries were eliminated, yielding a final analytical dataset of 821 unique publications. To resolve inconsistencies between WoSCC and PubMed in field format, author/institutional abbreviations, journal titles and year indexing rules, we supplemented a complete standardization workflow as follows:

1. All exported records from two databases were imported into EndNote 20 for initial duplicate screening using the built-in duplicate detection algorithm;
2. Two independent researchers manually cross-checked each record by title, first author, publication year, journal name and DOI. Discrepancies between the two screeners were resolved through group discussion. A total of 61 cross-database duplicate entries were eliminated, consistent with the number shown in the PRISMA flow diagram (**Figure 1**);
3. Uniform standardization of bibliographic fields: unified full names and abbreviations of affiliations, consistent capitalization and hyphen formats for author names, and standardized journal abbreviations compliant with ISO standards;
4. Unified time filtering threshold: only publications formally released between January 1, 2006 and December 31, 2025 were retained. Preprints and online advance publications without fixed volume/issue numbers were excluded to eliminate sample bias caused by inconsistent online indexing rules across the two databases.

The core innovation and central research objective of our work is to unpack the dynamic linkage between authoritative thyroid clinical guidelines (ATA, TI-RADS, Bethesda cytology criteria) and the clinical translation of AI diagnostic models, rather than conducting a general survey of artificial intelligence algorithms. PubMed serves as the authoritative biomedical database covering clinical ultrasound, endocrinology, and large-scale patient cohort validation studies, while Web of Science Core Collection systematically archives landmark clinical grading guidelines and clinically validated AI thyroid diagnostic research. The two databases together perfectly match our clinically oriented research positioning.

In contrast, engineering databases such as IEEE Xplore and ACM Digital Library primarily publish standalone algorithm simulation papers that lack real thyroid nodule clinical data, patient cohort verification, or alignment with standardized thyroid malignant risk stratification systems. Incorporating large volumes of purely computational papers without clinical validation would dilute our core thematic focus on guideline-driven AI clinical development and disrupt the interpretability of our bibliometric clustering and LDA topic modeling results.

This study analyzes only publicly available data from academic databases, involving no human participants, clinical specimens, or animal experiments; therefore, ethical approval and informed consent were not required.

### Data analysis and visualization

For the bibliometric analysis, all retrieved records were exported in plain text format, including author, title, source, citation, and abstract information, with the data selection process strictly adhering to Preferred Reporting Items for Systematic Reviews and Meta-Analyses (PRISMA) guidelines (45). Prior to analysis, data cleaning was performed to standardize discrepancies in author names, institutional affiliations, and country abbreviations, and duplicate entries were removed using EndNote followed by manual verification. To enhance methodological reliability and reduce subjective bias, two independent researchers conducted the literature screening, keyword clustering, and topic categorization, with disagreements resolved through discussion or consultation with a third reviewer; additionally, a random sample of 50 records was manually cross-checked to validate the accuracy of automated clustering and citation mapping (46, 47). Bibliometric and visualization analyses were then conducted using CiteSpace (version 6.1.R6) (48), VOSviewer (version 1.6.18) (28, 49), and an online bibliometric platform (24). Key attributes of the included articles were systematically extracted, with journal impact factors retrieved from the Journal Citation Reports and the h-index obtained from the Web of Science Core Collection (50). Publication output and growth trends were examined using the online platform. CiteSpace was employed for citation and co-citation analyses, collaboration network construction, citation burst detection, journal dual-map overlays, and keyword timeline clustering (51). Keyword clustering and timeline mapping in CiteSpace were performed using the log-likelihood2 algorithm to generate statistically robust thematic categorization. Meanwhile, VOSviewer was primarily utilized for reference clustering, with visual outputs illustrated as networks of nodes and links where colors denote distinct clusters (28). In this study, the node threshold for CiteSpace was set as Time slice = 1-year, Top N = 50, with pathfinder network pruning for sliced networks; VOSviewer adopted fractional counting for co-occurrence analysis. Two independent researchers completed literature screening with a Kappa consistency coefficient of 0.87 to reduce subjective bias. Conference abstracts, letters, editorials and case reports were strictly excluded to guarantee data homogeneity. The compound annual growth rate (CAGR) was calculated based on annual publication counts from 2006 (initial baseline volume) to 2025 (final full-year volume) using the standard formula:


$$CAGR={\left(\frac{Final\;annual\;output}{Initial\;annual\;output}\right)}^{\begin{array}{c} 1/n \end{array}}-1$$

where n denotes the number of observation years.

To explore the potential thematic structure of literature and clarify the research hotspots and semantic associations in the research field, this study adopts the LDA, a probabilistic generative unsupervised topic model, to conduct topic mining analysis based on literature abstracts. Without manual annotation, the LDA model can automatically excavate implicit semantics from large-scale texts, effectively compensating for the limitations of traditional text analysis methods that are highly subjective and incapable of capturing deep textual correlations. Thus, it is well-suited for literature topic clustering and content analysis. In this study, the LDA model is constructed and operated entirely using R language and the topicmodels package. The Gibbs sampling algorithm is applied for parameter estimation to continuously optimize model parameters through iterative sampling, which ensures the accuracy and stability of topic fitting for large-scale corpora. Candidate topic numbers ranged from 5 to 25 with an increment of 5. Perplexity and coherence scores were computed for each candidate k. The optimal topic number k=15 was selected based on the maximum coherence value alongside the elbow-like inflection of the perplexity curve. Hyper-parameters were set as document-topic prior α=0.05 and topic-term prior δ=0.1. Gibbs sampling was implemented with 1000 iterations and a burn-in period of 500 iterations. Prior to modeling, standardized text preprocessing is performed. Both unigram and bigram lexical features are extracted to enrich feature dimensions and incorporate both single-word semantics and phrase-based contextual associations. Meanwhile, multiple optimization procedures are implemented, including removing stop words, filtering low-frequency words with a word frequency less than 5, and eliminating high-frequency generic words above the 95th percentile of word frequency distribution. These practices purify the text corpus, streamline feature dimensions, and reduce the interference of sparse and generalized words on model fitting, thereby significantly improving the accuracy and distinguishability of LDA-based topic mining and laying a solid data and methodological foundation for subsequent thematic structure analysis.

## Results

### Publication of overview

**Figure 2:**
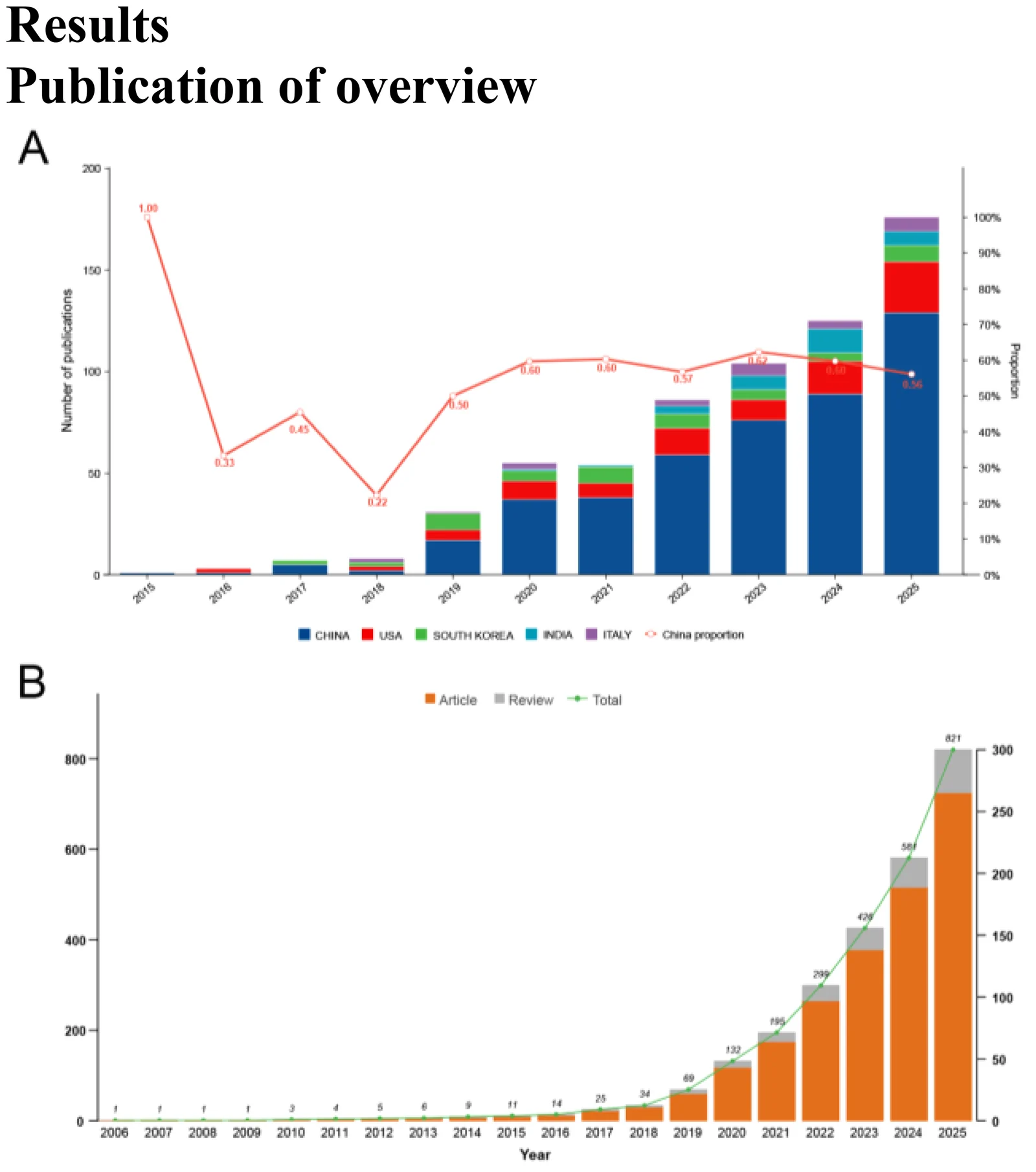
Annual publication output and country distribution. **(A)** Publication-output trends based on 821 valid records retrieved on June 1, 2026 (Beijing Time). The 240 publications recorded for 2025 represent all formally indexed full-calendar-year records from the WoSCC and PubMed databases, with no annual estimation or extrapolation. **(B)** Annual counts and growth trends for top 5 countries/regions.

From 2006 to 2025, after strict deduplication, filtering by document type (original article/review only), language, and exclusion of conference abstracts, letters, editorials, and case reports, the final analytic dataset included 821 valid publications (**Figure 1**). Annual publication trends were plotted based on these finalized 821 records. Fewer than 10 related papers were published per year before 2015, surpassing 10 papers in 2017, exceeding 60 papers after 2019, and stabilizing at over 100 papers annually since 2020(**Figure 2A**). Importantly, the final dataset for growth modeling and CAGR calculation comprised the 821 peer-reviewed publications from 2006 to 2025. The initial publication volume in 2006 was very low (fewer than 5 articles). The full-year indexed publication output reached 240 in 2025. This number corresponds to complete 2025 records indexed within two databases as of our unified retrieval snapshot (June 1, 2026, Beijing Time), without prediction or extrapolation, yielding a CAGR of 24.1% across the 2006–2025 period. Given the near-zero baseline in 2006, which may introduce calculation distortion, the CAGR was also verified using the stable growth phase from 2017 to 2025, confirming sustained rapid expansion (**Figure 2A**). At the country and regional level, **Figure 2B** presents the top five countries ranked by total publication output. China stands out as the leading contributor, with a substantial and steadily increasing volume of publications, followed by the United States, South Korea, Italy, and India, whose publication outputs have remained relatively stable. Importantly, India, as a developing country, has also demonstrated a significant growth trend in publication volume. In summary, research at the intersection of thyroid nodules and AI has entered a phase of rapid development since 2017, characterized by a sustained upward trajectory in annual publication output and a consistently high compound annual growth rate, indicating that this field is increasingly becoming a global academic hotspot.

### Analysis of national publications and collaborations

**Figure 3:**
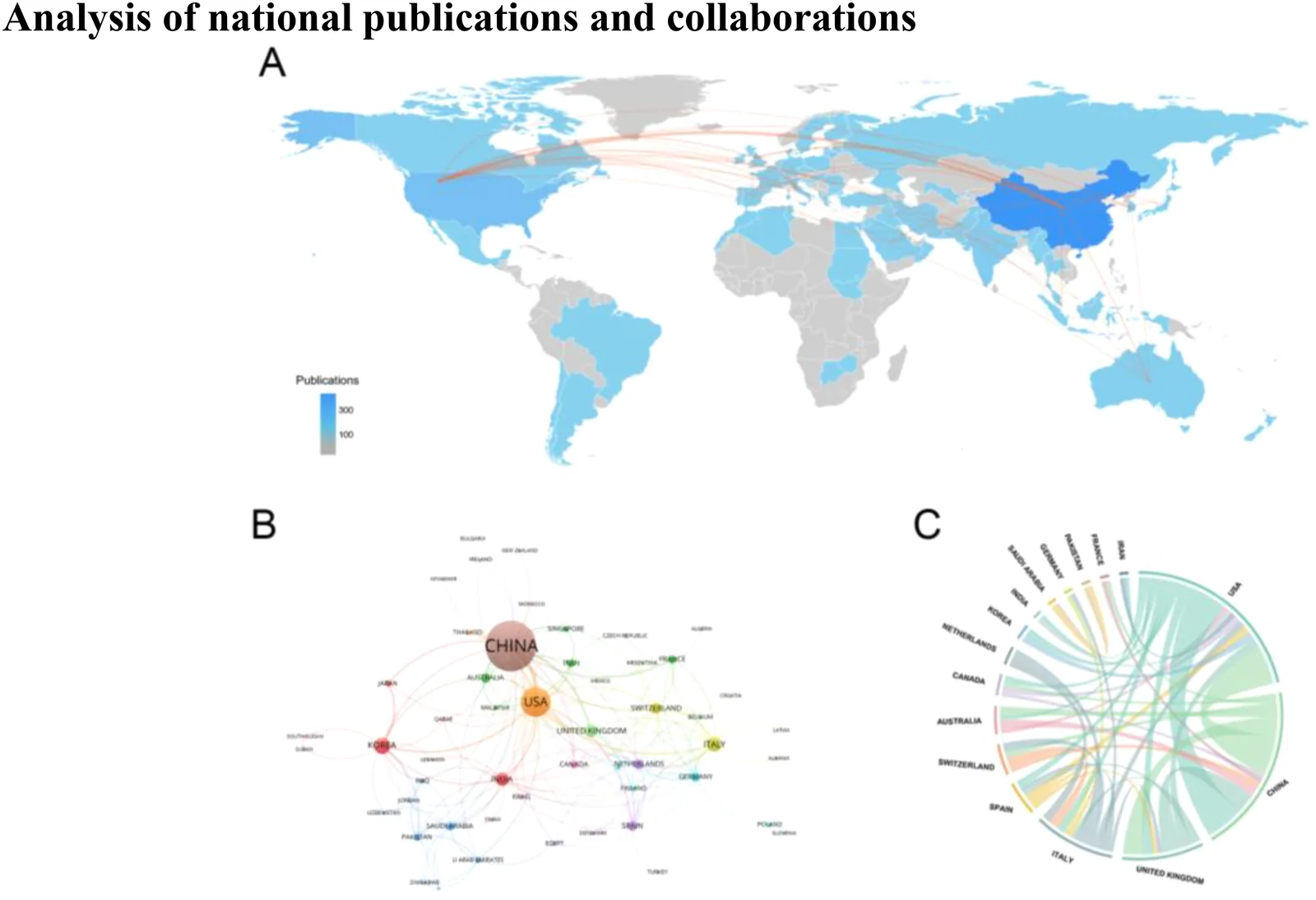
Geographic distribution and international cooperation. **(a)** Distribution of countries engaged in thyroid nodules and AI; **(b)** The network of co-country co-occurrence; **(c)** The network map of cooperation between countries or regions.

A total of 821 articles on thyroid nodules and artificial intelligence were contributed by 75 countries or regions. From a global geographical perspective, East Asia and North America are the most active research regions, with China, the United States, and South Korea forming three major research hubs. The research network among these countries shows high density, particularly frequent collaborations involving China, the US, South Korea, India, and Italy, indicating close international research cooperation (**Figure 3A**). **Table 1** lists the top 10 countries by publication volume. Together, these countries contributed 598 papers, accounting for 72.8% of all publications, reflecting a concentrated distribution pattern in this research field. The single-country publication (SCP) in China is 401, exceeding the total number of publications from other countries. Notably, China, as a developing country, dominates in total publications, share of articles, independent research, and international collaboration, with its output and citation counts far exceeding the sum of all other countries. Regarding international collaboration patterns, China’s multi-country collaboration publications (MCP) is 53, suggesting that independent research remains the mainstay. **Figure 3B**, the national collaboration network, further validates China’s academic leadership in the field. **Figure 3C** further shows that China serves as a core hub in global collaboration, cooperating with 21 countries and accounting for 24.7% of all international collaborations. The China-US partnership is the strongest bilateral collaboration, with a total of 25 co-authored papers. In summary, China plays a leading role in both research output and academic influence in this field, while international collaborations—particularly among core countries—are extensive and well-established.

**Table 1:** The top 10 countries of thyroid nodules and AI.

Country	Articles	Articles %	TC	Average article citations	SCP	MCP	MCP %
China	454	55.3	8,471	18.70	401	53	11.7
USA	91	11.1	2,418	26.60	69	22	24.2
Korea	49	6.0	1,449	29.60	40	9	18.4
India	33	4.0	539	20.00	29	4	12.1
Italy	27	3.3	396	66.00	18	9	33.3
Germany	12	1.5	287	8.70	9	3	25
Iran	11	1.3	165	15.00	9	2	18.2
Spain	11	1.3	161	13.40	8	3	27.3
Poland	9	1.1	130	16.20	8	1	11.1
United Kingdom	8	1.0	108	13.50	7	1	12.5

### Analysis of institutional publications

**Figure 4:**
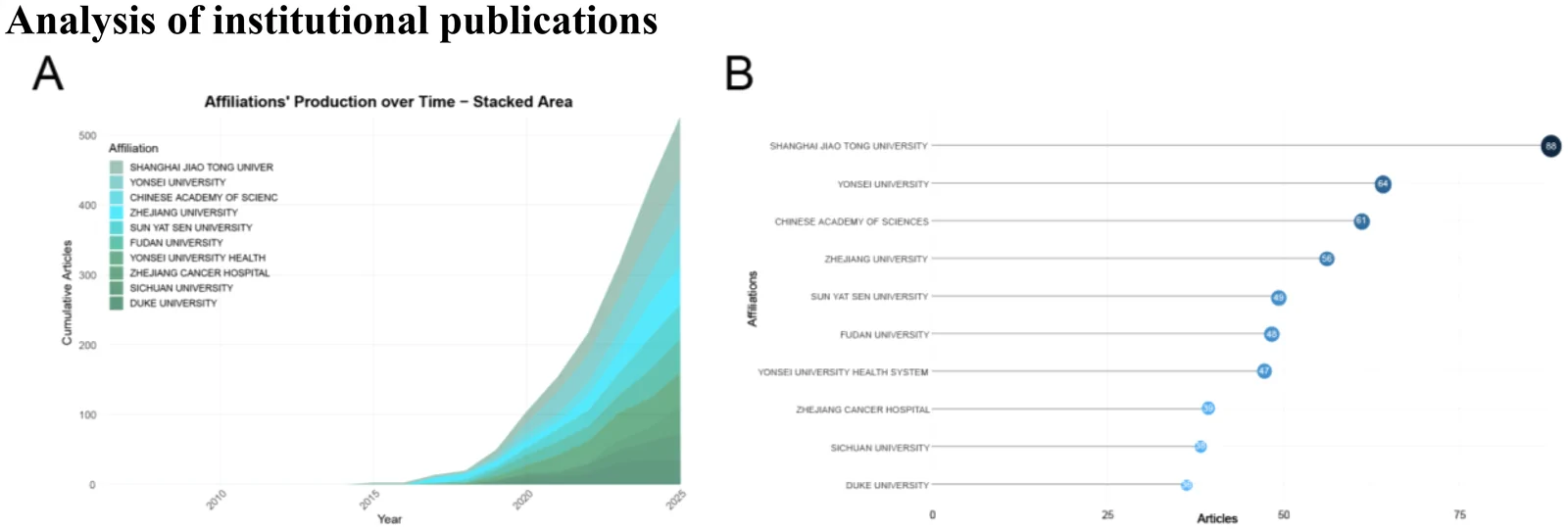
Institutional contribution and collaboration. **(A)** Trends in publication output of research institutions over time. **(B)** The top 10 most relevant research institutions currently active in the field of thyroid nodules and AI.

In terms of institutional publication output, Shanghai Jiao Tong University ranked first globally in cumulative publications, with its corresponding color block occupying the widest area at the top of the stacked area chart (**Figure 4A**). The Yonsei and Chinese Academy of Sciences University ranked second and third, respectively, both demonstrating interestingly accelerated growth after 2020, as reflected by the widening of their color blocks on the right side of the timeline. Meanwhile, other Chinese institutions, such as Zhejiang University and Sun Yat-sen University, also exhibited active performance, collectively highlighting the dominant role of Chinese institutions in total publication volume. From a temporal perspective, major institutions experienced rapid growth in publication output after 2017, consistent with the overall rapid development phase of this field (compound annual growth rate of 24.1%). As of 2025, Shanghai Jiao Tong University ranks first among universities in cumulative publications, with a total of 88 papers. It is followed by the Yonsei University (64 papers), University of Chinese Academy of Sciences (61 papers), and Zhejiang University (54 papers) (**Figure 4B**). Moreover, by centrality, Shanghai Jiao Tong University ranked first in both cumulative publication count and annual publication ranking. The institutions listed in the figures represent the top contributors by publication volume, underscoring their core role in the field of thyroid nodules and artificial intelligence research.

### Author analysis

**Figure 5:**
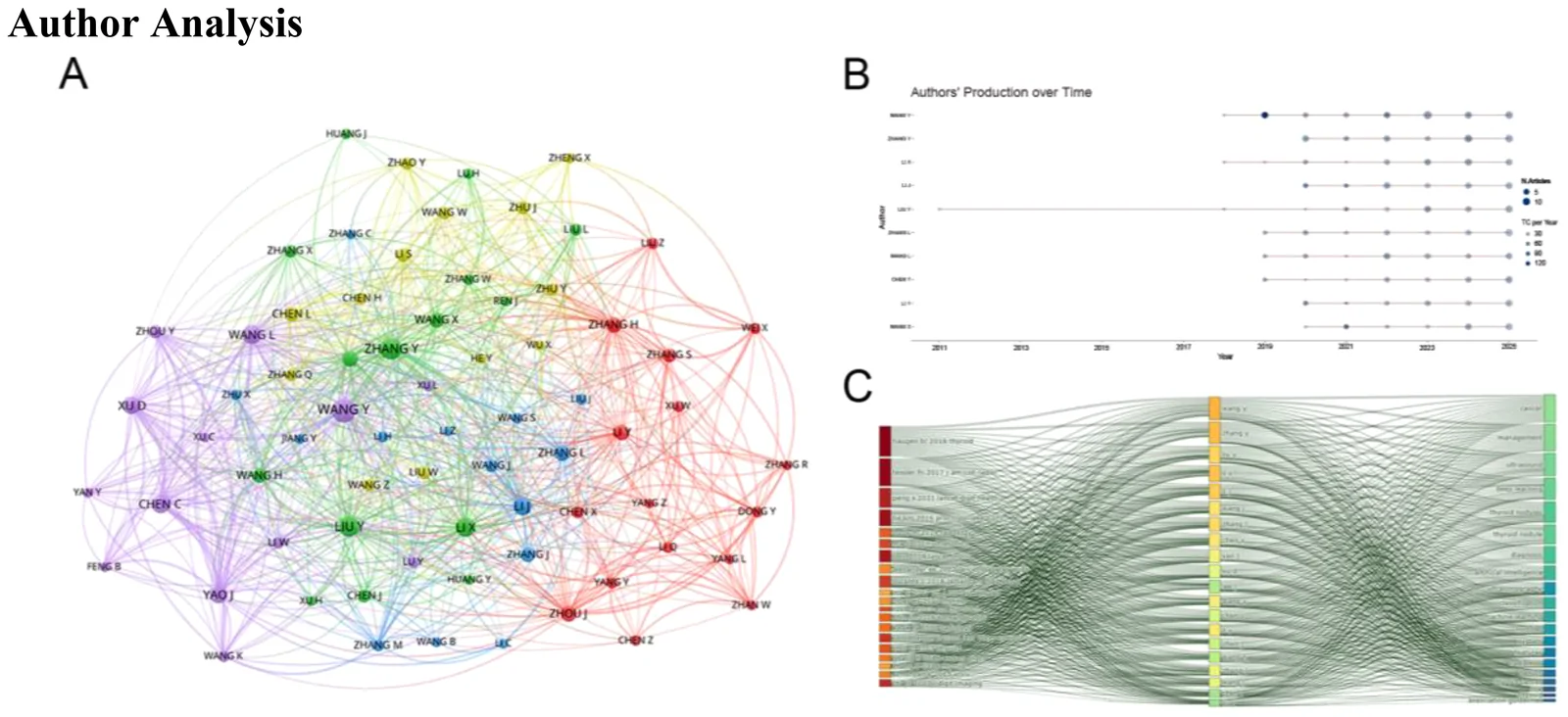
Author collaboration, productivity, and topic evolution. **(A)** The overlay map of coauthors in thyroid nodules and AI. **(B)** Authors’ production over time. **(C)** Topic evolution map analysis.

Integrating knowledge graphs with temporal trends, **Figure 5** presents a multidimensional analysis of collaborative relationships, academic influence, and research foci among authors in the field of thyroid nodules and AI. Based on co-authorship network patterns, 2870 authors were categorized into five distinct groups, differentiated by color. To examine high-frequency collaborations, authors with a minimum of three publications were selected, yielding a subset of 71 co-authors for the construction of an overlay visualization (**Figure 5A**). In this network, node size reflects the frequency of author co-occurrence, while connecting lines denote co-authorship relationships, collectively illustrating the structural configuration of collaborative patterns over time. Among the most influential contributors, WANG Y(National Clinical Research Center for Cancer), Zhang Y(Central South University), Liu Y(5th Clinical College of Inner Mongolia Medical University), and Li X(Tianjin University) dominated the co-citation network. Core teams led by these authors have driven major progress in AI ultrasound imaging and deep learning model validation for thyroid nodules. **Figure 5B** presents the top 10 authors by co-citation frequency; as of 2025, the highest number of co-citations is attributed to Wang Y, followed by Zhang Y, Li X, and Li J. This pattern is consistent with the structure of the collaboration network shown in **Figure 5A**. **Figure 5C** illustrates the continued growth in publication volume and citation impact among the leading authors, and further describes their research focuses through interrelationships among authors, highly cited articles, and keywords. The core academic teams led by Wang Y, followed by Zhang Y, Li X, and Li J, form the main collaborative clusters driving the mainstream research progress in artificial intelligence-based ultrasound for thyroid nodules and deep learning model validation. Overall, the network analysis in **Figure 5** systematically elucidates the intrinsic connections between prominent authors and their academic contributions.

### References analysis

**Figure 6:**
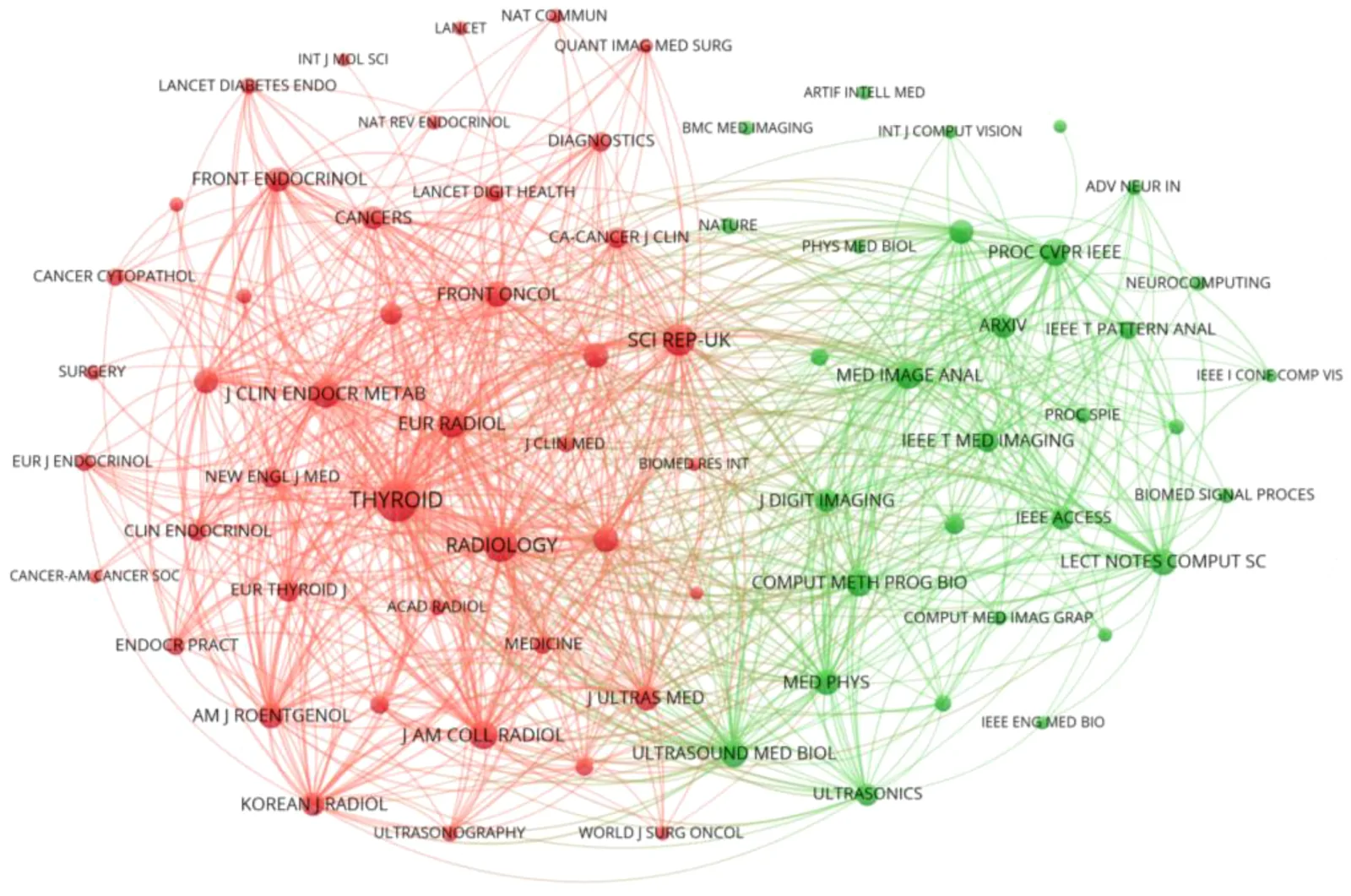
Journal distribution and citation analysis in thyroid nodules and AI.

VOSviewer was employed to analyze cited references, enabling the exploration of research distribution across different journals and providing valuable insights into academic publishing in the field of thyroid nodules and AI. The journal clustering visualization (**Figure 6**) illustrates the citation distribution among journals, with *Thyroid*, *Radiology* and *Sci Rep-UK* leading in terms of cited frequency, while the journal citation analysis highlights the core journals in this field, with *Engineering-Prc*, *J Digit Imaging* and *Jama Surg* occupying central positions in discussions on thyroid nodules and AI. **Table 2** lists the top ten journals by cited frequency; among them, *Engineering-Prc* ranked first in total citations (TC, 573) and annual average citations (71.63). Endocrine had the largest publication volume (20 articles) among the top journals, with TC of 287 and moderate annual citation intensity. This single high-citation article (Liu et al., 2018) is a landmark review focusing on AI medical imaging engineering, which accounts for its extremely outstanding citation intensity in this field (52). **Table 2** also presents detailed information on the top ten journals ranked by total publications, total citations, average citations per year, and impact factor. Notably, Endocrine published the highest number of articles among the top ten journals (20 articles) and accumulated 287 total citations; its total annual citation was 41 when calculated on a whole-journal basis, whereas the average citation per individual article was only 14.35. By contrast, Engineering-Prc included merely one landmark review paper focused on artificial intelligence in medical imaging engineering, which contributed all 573 cumulative citations of this journal and yielded an extraordinarily high per-article citation value, far exceeding the single-article citation level of Endocrine. Moreover, *Engineering-Prc* holds a high impact factor among these journals, further underscoring its significant influence and importance in the field of thyroid nodules and AI, particularly in terms of citation impact. In summary, the journal-level analysis reveals a diverse landscape of academic publishing, with *Engineering-Prc* demonstrating exceptional citation impact and *Endocrine* leading in publication volume, reflecting the multidisciplinary nature and growing scholarly attention in this field.

**Table 2:** Top 10 most productive journals related to thyroid nodules and AI.

Rank	Journal	Total citations	Article	TC per year	Normalized TC	Year	TC/ normalized TC)
1	Engineering-Prc	573	1	71.63	7.46	2019	9.6
2	J Digit Imaging	292	6	29.20	2.72	2017	10.7
3	Jama Surg	287	1	31.89	2.85	2018	11.2
4	Endocrine	287	20	41.00	7.37	2020	5.6
5	Lancet Digit Health	272	2	45.33	8.76	2021	5.2
6	Radiol-Artif Intell	244	1	48.80	12.18	2019	4.0
7	J Am Coll Radiol	244	2	30.50	3.18	2022	9.6
8	Ultrasonics	205	2	20.50	1.91	2017	10.7
9	Abdom Radiol	189	1	21.00	1.88	2017	11.2
10	Comput Meth Prog Bio	188	8	18.80	1.75	2018	10.7

### Highly cited references and citation burst analysis

**Figure 7:**
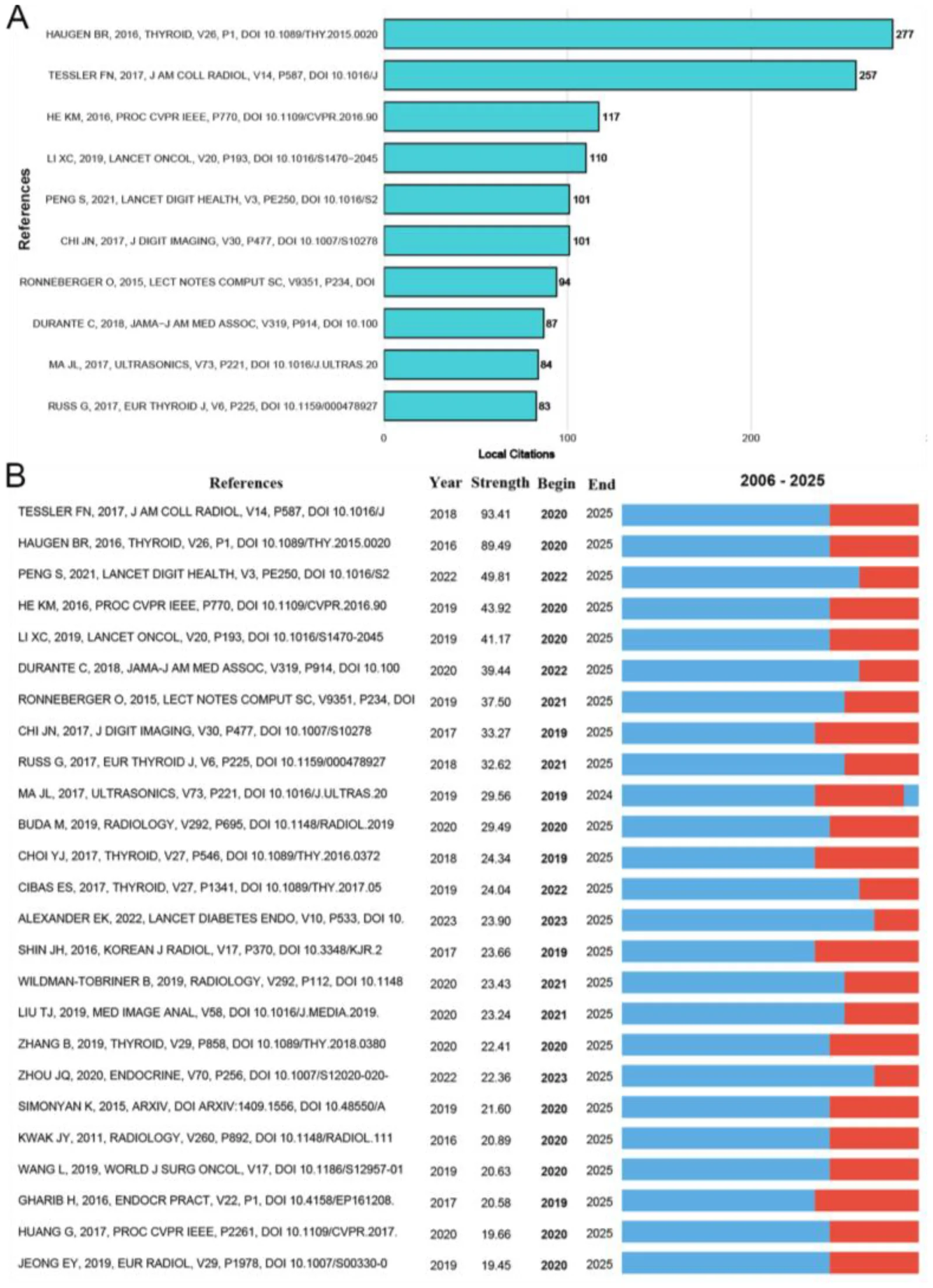
Analysis of highly cited references and citation bursts. **(A)** The top 10 most cited references in the field of AI thyroid nodules. **(B)** The top 20 references with the strongest citation bursts.

In the field of AI-based thyroid nodule research, the top 10 most-cited references accumulated a total of 1,326 citations, accounting for 10.4 % of the total citation pool within the final included bibliometric dataset (**Figure 7A**). The top highly cited references were dominated by classic clinical guidelines (ATA, TI-RADS, Bethesda), further proving that the overall AI research framework in this field has always been formulated and advanced following authoritative clinical diagnostic standards. Among them, the study by Haugen BR et al., "2015 American Thyroid Association Management Guidelines for Adult Patients with Thyroid Nodules and Differentiated Thyroid Cancer: The American Thyroid Association Guidelines Task Force on Thyroid Nodules and Differentiated Thyroid Cancer" published in *Thyroid* in 2016, has accumulated 277 total citations, with an average of 31 citations per year, highlighting its substantial influence (6); meanwhile, the work by Tessler FN et al., "ACR Thyroid Imaging, Reporting and Data System (TI-RADS): White Paper of the ACR TI-RADS Committee," published in the *Journal of the American College of Radiology* in 2017, received 257 total citations, also averaging 32 citations annually, further underscoring its significance in the field (7). In terms of citation bursts, the study by Tessler F et al., published in the *Journal of the American College of Radiology* in 2017, exhibited the highest burst strength (93.41, normalized citation burst value detected by CiteSpace), with its peak period spanning from 2020 to 2025 (7); interestingly, four publications continue to show ongoing citation bursts, focusing respectively on *convolutional networks for biomedical images (*53), *ultrasound malignant risk stratification guidelines for adult thyroid nodules diagnosis (*8), *diagnosis of thyroid nodules *(9) and *the bethesda system for reporting thyroid cytopathology (*10). These persistent bursts reflect sustained academic interest in early diagnosis, screening effectiveness, and the global epidemiology of thyroid cancer, further reinforcing the interdisciplinary value of AI in public health and clinical research (**Figure 7B**). In summary, these highly cited and persistently impactful studies underscore the growing convergence of AI with thyroid nodule research, particularly in enhancing diagnostic accuracy, standardization, and public health applications.

### Keyword analysis

**Figure 8:**
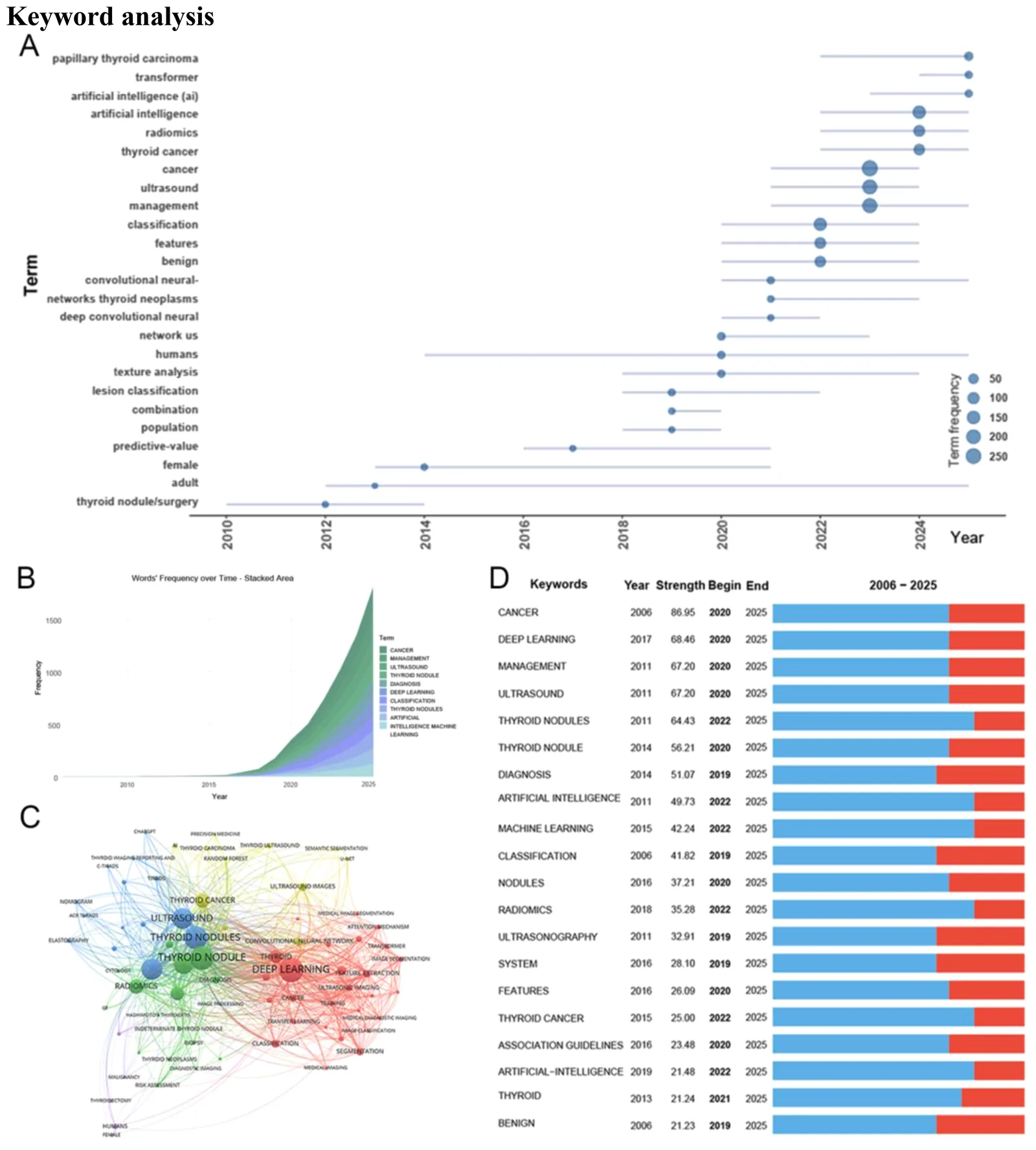
Keyword analysis and evolving trends. **(A)** Timeline diagram of reference co-citation analysis. **(B)** The frequency of keywords changes over time. **(C)** Keyword clustering diagram. **(D)** Top 20 keywords with the strongest citation bursts.

Based on the existing keyword timeline and citation burst results in **Figure 8**, obvious chronological consistency was observed with mainstream clinical guideline updates: the rapid surge of machine learning and risk stratification hotspots emerged gradually after the release of the 2015 ATA management guidelines; the prominent burst of convolutional neural network (CNN), deep learning and ultrasound arose following the widespread adoption of ACR TI-RADS, EU-TIRADS and the Bethesda system in 2017; after 2021, the emerging bursts of multimodal, explainable artificial intelligence (XAI) and radiomics further reflected that the latest iterative optimization of clinical grading requirements promoted the transformation of AI research from simple image recognition to refined multimodal auxiliary diagnosis.

Keywords serve as labels for research content, effectively revealing emerging hotspots and evolving trends. We conducted a timeline analysis of keywords, in which different colors represent distinct keyword clusters, and the area of each block reflects the frequency of keyword occurrence. The analysis showed that "cancer", "ultrasound" and "management" were the most prominent keywords (**Figure 8A**). **Figure 8B** showed the trends in the frequency of major keywords over time. The summed occurrence frequency of four core keywords rose from approximately 200 in 2020 to 1100 in 2025, corresponding to a compound annual growth rate of 40.6%, indicating sustained growing research interest in these core topics. **Figure 8C** presents the visualization of the keyword clustering analysis, where node size indicates keyword frequency and the distance between nodes reflects the closeness of their associations. Keywords located close to one another are grouped into clusters, representing the primary research foci in the field of thyroid nodules and AI. These clusters can be broadly categorized into three groups. The first cluster, represented in blue, includes keywords such as thyroid nodules, ultrasound, and AI; the second cluster, shown in red, comprises deep learning, cancer, and thyroid; and the third cluster, in green, includes THYROID NODULES, machine learning, and ULTRASONOGR. **Figure 8D** presents the top 20 references with the strongest citation bursts, with the highest burst strength reaching 86.95. The results revealed that "cancer" had the longest burst duration. In recent years, "cancer", "deep learning" and "management" have become the most prominent research topics. These sustained bursts reflect continued interest in the early diagnosis of thyroid nodules, screening effectiveness, and global epidemiology, further underscoring the importance of interdisciplinary applications of AI in public health and clinical research. The sustained burst of "cancer" reflects the core demand of clinical malignant risk screening, which is the fundamental driving force of AI application in thyroid nodules.

### LDA-based topic modeling

**Figure 9:**
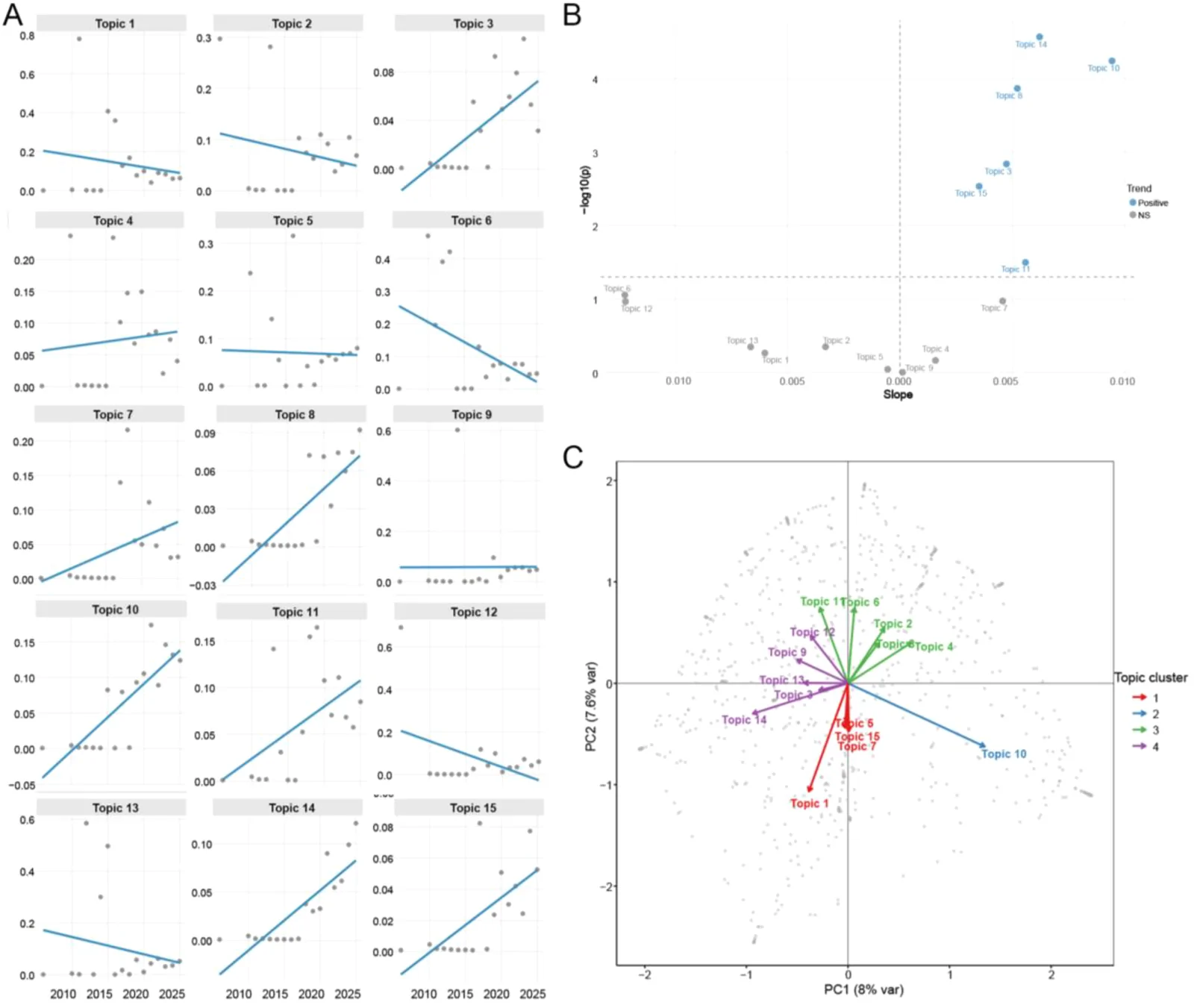
Topic dynamics and document-topic structure. **(A)** shows the temporal trends (2006-2025) of latent topics in AI-based thyroid nodule research, presenting the annual posterior probabilities of 15 topics (Topic 1 to Topic 15), including observed values (points) per year and fitted linear trends. The direction and magnitude of the slopes indicate the rise or decline in topic attention over time. **(B)** is a volcano plot visualizing the slope and statistical significance of temporal trends for individual topics. **(C)** is a Husson−Jongmans biplot illustrating the document−topic distribution based on principal component analysis of the topic posterior matrix (θ). Four topic clusters (Cluster 1 to Cluster 4; colors as shown in the legend) are overlaid in this biplot to highlight topic groupings and document−topic relationships.

We applied LDA to the corpus (excluding any records without abstracts) and identified 15 major latent topics. After manually reviewing the top 20 terms and representative literatures for each topic, we labeled the topics accordingly. Prominent topics included Topic 10: "Classification Methods" (N = 105; 12.17%), Topic 14: "Deep Learning" (N = 64; 8.17%), Topic 1: "Pathological Judgment" (N = 62; 7.61%), and Topic 2: "AI Diagnosis" (N = 62; 7.38%) (**Table 3**). In Topic 8 (Imaging Technology) and Topic 11 (Thyroid Nodule Assessment), notable terms including “ChatGPT”, “Claude”, and “Gemini” were observed within the topic-term matrix. These terms originate from exploratory publications published between 2024 and 2025. Such emerging studies investigate the application of large-language models for clinical decision-support in thyroid nodule management, focusing on prompt engineering and large language model(LLM)-assisted diagnostic reasoning. This nascent sub-theme aligns with the research frontier of XAI identified in the present work, representing a newly-emerging exploratory research direction within this domain. To examine temporal dynamics, we fit a linear regression for each topic, using publication year as the independent variable and the annual average posterior topic probability as the dependent variable. Trends were classified based on the slope and statistical significance (threshold: P< 0.05). **Figure 9A** shows topics with significant positive trends, including Topic 14 (Deep Learning; slope = 0.0062, P< 0.0001), Topic 10 (Classification Methods; slope = 0.0094, P< 0.0001), Topic 8 (Imaging Technology; slope = 0.0052, P = 0.0001), Topic 3 (AI Automatic Diagnosis; slope = 0.0047, P = 0.0015), Topic 15 (Ultrasound-Based Risk; slope = 0.0035, P = 0.0029), and Topic 11 (Thyroid Nodule Assessment; slope = 0.0056, P = 0.0316), indicating increasing attention to these topics. The remaining topics showed no significant temporal changes (P > 0.05), suggesting relative stability. These upward trends were further highlighted in a volcano plot (slope vs -log10P) (**Figure 9B**). To make the above logic coherent: Principal component analysis (PCA) was performed on the document-topic distribution matrix θ, and the results were visualized using a Husson-Jongmans biplot. The first two principal components explained approximately 8.0 % LDA-based topic mining (PC1) and 7.6 % (PC2) of the variance, respectively. In the biplot, gray points represent individual documents, while colored arrows denote topic vectors: longer arrows indicate stronger contributions to the respective principal components, and documents located in the direction of an arrow have higher posterior probabilities for that topic. Four main clusters emerged: Cluster 1 (lower side) encompasses studies related to AI-assisted pathological diagnosis of thyroid nodules, such as B-Mode Ultrasound and Machine Learning; Cluster 2 (right region) focuses on methodological aspects of diagnostic grading; Cluster 3 (upper region) includes characteristics related to AI-diagnosis; Cluster 4 (negative PC1 axis) emphasizes AI automatic diagnosis, including terms like AI Automatic Diagnosis and diagnostic accuracy (**Figure 9C**). The topics formed four clusters (Clusters 1-4; colors as shown in the legend), highlighting topic groupings and document-topic relationships. The relatively low cumulative explained variance of the first two principal components is mainly attributed to high topic heterogeneity across thyroid AI research and scattered text features derived from heterogeneous abstract contents.

**Table 3:** Topics discovered from 821 articles published between 2006 and 2025.

Topic	Prevalence	Top terms	Label	N	Representative article title
Topic 10	0.12	classification, adopts, atrous, boost, cdus, competitive, constrained, crucial role, datasets demonstrate, ddti dataset	Classification Methods	105	Prediction Multiscale Cross-Level Fusion U-Net with Combined Wavelet Convolutions for Thyroid Nodule Segmentation
Topic 14	0.08	deep learning,radiomics, ai ceus, auc calibration, aucs training, brier, category thyroid, cstn, demonstrated best, demonstrated excellent, dtl	Deep Learning	64	Enhancing diagnostic precision for thyroid C-TIRADS category 4 nodules: a hybrid deep learning and machine learning model integrating grayscale and elastographic ultrasound features
Topic 1	0.08	judgment, malignancy scores, c-rsna, cutoff value, cytopathologic, expert consensus, fnabs, forward, iv v, scanner	Pathological Judgment	62	An Artificial Intelligence Model Based on ACR TI-RADS Characteristics for US Diagnosis of Thyroid Nodules
Topic 2	0.07	ai-diagnosis, ai-transformer for thyroid, algorithms based, application deep, approach based, autop, consuming, fnd, institute, nuclei	Ai-diagnosis	62	Artificial Intelligence for Evaluation of Thyroid Nodules: A Primer
Topic 11	0.08	thyroid nodules, auroc curve, ci pooled, differ significantly, final histopathology, gemini claude, guidelines best, head neck, less experienced, mpus	Thyroid Nodule Assessment	61	Effectiveness evaluation of computer-aided diagnosis system for the diagnosis of thyroid nodules on ultrasound A systematic review and meta-analysis
Topic 8	0.07	adversarial networks, chatgpto, claude opus, high specificity, language models, language processing, learning thyroid, madlap, natural language, opus	Imaging Technology	58	Improving diagnostic precision in thyroid nodule segmentation from ultrasound images with a self-attention mechanism-based Swin U-Net model
Topic 5	0.07	cytological diagnosis, focal, format, gpt model, magnetic, magnetic resonance, metabolism, microscopic, poverty	B-Mode Ultrasound	56	Multiparametric ultrasound evaluation of thyroid nodules
Topic 4	0.06	B-mode Ultrasound, alexnet, busceus radiomics, continuously, database tdid, dataset dataset, dlrt, geometry, model ceus, pixellevel labels	Ultrasound Features	56	Segmentation of thyroid nodules from ultrasound images using convolutional neural network architectures
Topic 3	0.06	ai automatic, auroc values, calcified, deficiency, gradientweighted, gradientweighted class, half, hypoechogenicity, hypothyroidism goiter, iodine deficiency	AI Automatic Diagnosis	51	CAD system based on B-mode and color Doppler sonographic features may predict if a thyroid nodule is hot or cold
Topic 6	0.06	medical features, medicine biology, biopsy center, commercially, commercially available, contributing, department, exclusion, federation, federation ultrasound	Medical Features	49	Deep learning-based segmentation of nodules in thyroid ultrasound: improving performance by utilizing markers present in the images
Topic 7	0.05	aucroc, causes, clinics, comfort, degenerative, developed deep, fpr, illustration, improve quality, learning classification	Machine Learning Classification	43	A Novel Deep-Learning-Based CADx Architecture for Classification of Thyroid Nodules Using Ultrasound Images
Topic 9	0.05	auxiliary diagnosis, clnm ptc, ct value, dlct quantitative, elderly, endometriosis, insulin, kev, model, nodule imaging	Auxiliary Diagnosis	41	Identification of benign and malignant thyroid nodules based on dynamic AI ultrasound intelligent auxiliary diagnosis system
Topic 15	0.05	fna biopsies, image similarity, nmtn-mmtn, ultrasound-based risk, use package amsbsy	Ultrasound-Based Risk	40	AIBx, Artificial Intelligence Model to Risk Stratify Thyroid Nodules
Topic 12	0.05	accuracy risk, cl-sic, different us, distinctive, ffdgpositive, immune, indications, kwaktirads, mtntirads, nlr	Risk Accuracy	35	Ultrasound Risk Stratification of Autonomously Functioning Thyroid Nodules: Cine Loop Video Sequences Versus Static Image Captures
Topic 13	0.04	thyroid, cohort independent, combined gene, deep radiomics, multicenter cohort, prospective multicenter, svmlin, svmrbf,	Thyroid multicenter modeling	35	A novel approach to quantify calcifications of thyroid nodules in US images based on deep learning: predicting the risk of cervical lymph node metastasis in papillary thyroid cancer patients

## Discussion

This bibliometric analysis provides a systematic overview of the global research landscape concerning AI applications in thyroid nodule diagnosis, covering two decades from 2006 to 2025. By integrating quantitative methods with visualization tools, we have delineated the evolutionary trajectory, core research themes, and collaborative networks that define this rapidly expanding field. The field achieves a compound annual growth rate of 24.1% with a distinct inflection point in 2017, signifying the rapid incorporation of artificial intelligence into thyroid nodule diagnosis. This rapid expansion may be partly attributable to multiple converging factors, including global technical maturation of CNN-based ultrasound algorithms and increased research funding in several countries, such as China's 2017 New Generation Artificial Intelligence Development Plan. Meanwhile, widespread clinical adoption of thyroid ultrasound screening in China’s tertiary and secondary hospitals has yielded large-scale, standardized domestic ultrasound datasets of thyroid nodules. This has reduced data access barriers for deep learning model training and may have contributed to the sharp rise in regional research output.

China contributes more than half of all included publications, yet the US shows higher average citations per paper, which reflects citation impact rather than an intrinsic measure of research quality. This disparity may be partly explained by several contextual considerations. First, national top-down AI investment prioritizes domestic medical imaging innovation and supports large-scale single-center ultrasound cohort construction; second, universal thyroid ultrasound screening policy in China creates abundant real-world clinical data unavailable in many Western countries; third, Chinese researchers actively localized ATA/TI-RADS risk stratification criteria for ethnic thyroid nodule morphological features, forming a sustained domestic research niche that drives high publication volume. By contrast, a relatively larger proportion of US-affiliated publications are associated with prospective cohort designs, which may contribute to their higher citation rates, though this pattern reflects research design choices rather than superiority in research quality. Cross-border Sino-US collaborations remain the strongest bilateral partnership, serving to bridge large Asian datasets and rigorous Western clinical validation frameworks. Such collaborations are essential for fostering methodological innovation and enabling large-scale, multicenter validation of AI models, which are critical for clinical translation.

Shanghai Jiao Tong University ranks first globally in cumulative publications, alongside a cluster of high-output Chinese comprehensive universities. Its high productivity may be associated with integrated cross-disciplinary platforms and sustained institutional funding support. The geographic concentration of high-output institutions may raise concerns about the generalizability of AI models to populations with different demographic and clinical characteristics: AI models trained exclusively on cohorts from resource-sufficient populations may fail to generalize to low- and middle-income regions with limited ultrasound equipment, uneven screening coverage, and distinct ethnic thyroid lesion characteristics.

Journal analysis reveals a diverse publishing landscape, with *Engineering-Prc* demonstrating the highest citation impact despite publishing only one article, while *Endocrine* leads in publication volume. The presence of high-impact journals such as *Lancet Digital Health* and *Radiology: AI* among the top-cited sources underscores the growing recognition of AI applications in clinical endocrinology and medical imaging. The multidisciplinary nature of these journals reflects the convergence of computer science, radiology, and endocrinology-a hallmark of this research field.

Beyond citation and keyword-based bibliometric outcomes, **Figure 9B** topic modeling further quantitatively unpacks the implicit thematic evolution hidden in massive abstract texts of this field. Fifteen latent research topics were identified after optimizing topic quantity by coherence and perplexity indicators, among which six topics including deep learning algorithm construction, nodule classification modeling and ultrasound imaging assessment presented statistically significant rising trends along the study period, consistent with the growth tendency of core keywords obtained from bibliometric clustering analysis. The PCA-based Husson-Jongmans biplot further divided all research themes into four differentiated clusters corresponding to pathological diagnosis research, grading methodological exploration, general AI diagnostic development and automatic auxiliary modeling, systematically revealing the inner disciplinary differentiation of thyroid AI research. Compared with conventional bibliometric clustering limited to keyword co-occurrence, LDA complements invisible text-level thematic information and avoids bias caused by single-index keyword statistics, which is an important methodological innovation distinguishing our research from previous pure bibliometric reports. Nevertheless, partial topics such as Topic 15 (Ultrasound-Based Risk) exhibited a statistically significant yet modest rising trend, reflecting scattered exploratory attempts in this peripheral direction, while malignant-risk diagnosis remains the core research priority of this field.

Keyword and citation burst analyses further elucidate the intellectual structure of the field. Dominant themes such as “deep learning,” “ultrasound,” and “cancer” highlight the central role of imaging-based AI in malignant risk stratification. The sustained burst of terms such as “management” and “diagnosis” indicates ongoing efforts to integrate AI into clinical workflows. Of note, the persistent citation bursts of foundational guidelines-such as the ATA and ACR TI-RADS—suggest that AI research remains closely tethered to established clinical frameworks, with emerging efforts focused on enhancing, rather than replacing, existing risk stratification systems. This chronological coupling relationship between guideline updates and research hotspot transformation is the core innovative finding of the present research: the release of the 2015 ATA guideline was followed by a surge in machine learning-based risk stratification research; the widespread adoption of ACR TI-RADS together with Bethesda cytology criteria in 2017 coincided with the explosive development of CNN and deep learning for ultrasound interpretation; and the updated refined grading standards after 2021 were temporally associated with the shift of AI exploration toward multimodal fusion and XAI. It should be noted that these observations reflect temporal correspondences rather than causal relationships; the identified associations do not imply that guideline updates directly caused shifts in AI research trajectories, but rather suggest a chronological alignment that warrants further investigation.

The high citation frequency of key deep learning methodologies and imaging guidelines reflects the field’s reliance on robust clinical benchmarks. In parallel, the increasing prominence of terms such as “radiomics” and “convolutional neural network” signals a shift toward more sophisticated feature extraction and model architectures. These trends align with broader movements in medical AI toward explainability, multimodal integration, and prospective clinical validation.

Collectively, the results of this study provide clear and actionable clinical translational guidance for artificial intelligence-assisted diagnosis of thyroid nodules based on current research hotspots. First, the guideline-driven evolutionary pattern verified in this study demonstrates that future AI model development and clinical validation should prioritize alignment with authoritative ATA, TI-RADS, and Bethesda grading criteria, rather than pursuing superficial algorithmic performance gains. This guideline-consistent optimization can unify AI diagnostic standards across medical centers and substantially promote standardized large-scale clinical implementation. Second, the clarified phased developmental trajectory bridges technical innovation and unmet clinical demands, facilitating the transformation of thyroid AI research from theoretical algorithm exploration to standardized, routine clinical auxiliary diagnosis.

In summary, this bibliometric analysis not only maps the current landscape of AI in thyroid nodule research but also reveals the structural factors affecting its evolution, including geography, institutional settings and research themes. By identifying leading contributors, influential works, and emerging frontiers, our findings provide a valuable reference for researchers seeking to navigate this dynamic field and identify opportunities for impactful investigation. To address these unmet translational barriers, three pivotal emerging research frontiers have emerged, each targeting specific clinical pain points in real-world AI deployment. Three distinct emerging research frontiers each directly address well-documented clinical bottlenecks hindering real-world AI clinical deployment. First, XAI tackles the critical opacity flaw of conventional black-box deep learning models, which fail to deliver transparent feature attribution and thus discourage radiologists and endocrinologists from trusting automated malignant risk stratification to guide biopsy decisions; XAI pipelines overcome this limitation by visualizing ultrasound morphological features matched to TI-RADS standards, boosting clinician acceptance and satisfying regulatory interpretability requirements. Second, multimodal data fusion mitigates the constrained diagnostic performance of standalone B-mode ultrasound for indeterminate thyroid lesions: combining ultrasound scans with fine-needle aspiration molecular signatures, thyroid serological indicators and genomic profiles breaks the accuracy bottleneck and supports personalized risk evaluation compliant with updated ATA clinical guidelines. Third, standardized multicenter prospective validation resolves the poor cross-hospital generalizability plaguing most existing algorithms, which are solely trained on single-center retrospective cohorts; large-scale prospective trials unified under ACR TI-RADS grading systems generate robust high-level clinical evidence that is a prerequisite for integrating AI tools into routine diagnostic guidelines. Taken together, these research avenues are not merely discrete algorithmic refinements, but purpose-built interventions targeting three fundamental translational obstacles: insufficient model interpretability, diagnostic limitations of unimodal imaging, and a scarcity of high-quality prospective clinical validation evidence. Future developmental trajectories will continue to follow iterative updates of authoritative thyroid diagnostic criteria, advancing standardized AI model optimization, multimodal intelligent integration, and large-scale multicenter clinical validation. Collectively, these efforts will accelerate high-quality clinical translation of AI-assisted thyroid diagnostic tools and foster precision, standardized endocrine care.

## Limitations

Several limitations should be considered when interpreting the findings of this study. First, literature was retrieved from two authoritative databases (WoSCC and PubMed) with only English articles included; Chinese core databases such as CNKI and Wanfang were not integrated, which may omit localized clinical research data from East Asia. In addition, preprint manuscripts, graduate dissertations and other grey literatures indexed in non-core academic repositories were not retrieved, which may omit emerging preliminary research outcomes.

Second, this bibliometric analysis focused on publication trends and citation characteristics, without quantitative evaluation of AI model performance (AUC, accuracy, specificity), which needs further systematic review in subsequent clinical meta-analysis.

Third, bibliometric indicators such as citation counts and h-indices are inherently time-dependent and may favor older publications, which have had more time to accumulate citations. Although citation burst analysis partially addresses this limitation by identifying temporally concentrated attention, it does not fully capture the impact of recent high-quality studies that have not yet had sufficient time to gain visibility.

Fourth, while data cleaning and manual verification were performed to minimize errors, issues such as author name disambiguation, institutional name variations, and inconsistent country affiliations remain inherent challenges in bibliometric analysis. Despite efforts to standardize these elements, some degree of residual inaccuracy is unavoidable.

Fifth, this bibliometric analysis only retrieved records from WoSCC and PubMed, without integrating mainstream computer engineering repositories including IEEE Xplore, ACM Digital Library, and the engineering sub-library of ScienceDirect. This strategy inevitably excludes influential pure deep learning methodological papers published in computer conferences and specialized engineering journals. Most of these excluded algorithmic studies have not been validated under real thyroid diagnostic workflows, and thus cannot support our core finding regarding the iterative interplay between clinical diagnostic standards and AI-assisted diagnosis research. Even so, the absence of engineering literature creates incomplete coverage of the computational development branch within this interdisciplinary field. Future related meta-research should combine both medical and engineering databases to mitigate this bias.

Sixth, linear regression was applied to assess temporal trends for 15 LDA-derived topics in the present study, yet no multiple-testing adjustment was performed for these exploratory analyses. As such, the reported P-values for topic-level temporal trends should be interpreted with caution. Multiple-testing correction will be adopted in future related research to enhance the robustness of statistical inference.

Finally, this study focuses on quantitative patterns of publication activity and citation impact, which do not necessarily reflect the clinical relevance, methodological rigor, or real-world utility of the analyzed research. Future studies could complement bibliometric findings with qualitative assessments, such as systematic reviews or meta-analyses, to provide a more comprehensive evaluation of the field.

## Conclusion

This comprehensive bibliometric analysis systematically analyzed 821 eligible publications concerning artificial intelligence applications in thyroid nodule diagnosis over a two-decade period (2006-2025), incorporating preliminary 2025 literature to update the latest research progress. Quantitatively, the field has witnessed sustained and rapid development, with a compound annual growth rate of 24.1%. The year 2017 marked a critical inflection point, after which annual publication output entered a continuous explosive growth stage, indicating that AI-assisted thyroid nodule diagnosis has evolved into a mainstream interdisciplinary research hotspot. In terms of global research patterns, China dominates the field in publication volume and independent research output, while the United States and South Korea exhibit superior citation quality and academic influence. High-productivity institutions are primarily Chinese universities, led by Shanghai Jiao Tong University, constituting intensive and cooperative global academic networks at both national and institutional levels. Thematically, core research hotspots focus on ultrasound imaging analysis, deep learning algorithm construction, and malignant risk stratification of thyroid nodules, and our core innovative discovery clarifies the temporal association between sequential iteration of ATA, TI-RADS and Bethesda clinical criteria and the phased evolution of deep learning and radiomics research. Differing from prior studies relying on shorter research windows, single database retrieval and limited sample size, this work built a 20-year long-time-series framework based on dual-database retrieval and standardized data cleaning of 821 literature records; additionally, supplementary LDA topic modeling broke through the limitation of traditional bibliometrics, realizing quantitative mining of implicit thematic structure from abstract texts and completing multidimensional field knowledge mapping. Emerging frontiers further point to the future development orientation of XAI, multimodal data fusion, and standardized large-scale multicenter clinical validation. On this basis, this study supplements a feasible multi-database retrieval paradigm for subsequent relevant research. Future bibliometric investigations into AI for thyroid nodules may simultaneously integrate WoSCC, PubMed and IEEE Xplore datasets to construct a dual-dimensional research database that covers both clinical translational research and pure algorithm engineering research domains. Comparative analysis of thematic evolution between clinically validated diagnostic studies and independent computational modeling works is expected to yield a more holistic and comprehensive panorama of the entire research field. This study comprehensively maps the knowledge structure, evolutionary trajectory, and research prospects of AI applications in thyroid nodule diagnosis. It provides credible references for subsequent algorithm optimization, clinical translational research, and standardized clinical practice, and further promotes the high-quality development of AI-assisted diagnostic technology and precision endocrinology.

## Data Availability

The original contributions presented in the study are included in the article/supplementary material. Further inquiries can be directed to the corresponding author.
